# Metabolic engineering of *Escherichia coli* for enhanced arginine biosynthesis

**DOI:** 10.1186/s12934-015-0211-y

**Published:** 2015-03-07

**Authors:** Mireille Ginesy, Jaroslav Belotserkovsky, Josefine Enman, Leif Isaksson, Ulrika Rova

**Affiliations:** Biochemical Process Engineering, Division of Chemical Engineering, Department of Civil, Environmental and Natural Resources Engineering, Luleå University of Technology, SE-971 87 Luleå, Sweden; Department of Molecular Biosciences Wenner-Gren institute, Stockholm University, SE-106 91 Stockholm, Sweden

**Keywords:** *Escherichia coli*, L-arginine, Metabolic engineering, Fermentation

## Abstract

**Background:**

Arginine is a high-value product, especially for the pharmaceutical industry. Growing demand for environmental-friendly and traceable products have stressed the need for microbial production of this amino acid. Therefore, the aim of this study was to improve arginine production in *Escherichia coli* by metabolic engineering and to establish a fermentation process in 1-L bioreactor scale to evaluate the different mutants.

**Results:**

Firstly, *argR* (encoding an arginine responsive repressor protein), *speC*, *speF* (encoding ornithine decarboxylases) and *adiA* (encoding an arginine decarboxylase) were knocked out and the feedback-resistant *argA214* or *argA215* were introduced into the strain. Three glutamate independent mutants were assessed in bioreactors. Unlike the parent strain, which did not excrete any arginine during glucose fermentation, the constructs produced between 1.94 and 3.03 g/L arginine. Next, wild type *argA* was deleted and the gene copy number of *argA214* was raised, resulting in a slight increase in arginine production (4.11 g/L) but causing most of the carbon flow to be redirected toward acetate. The V216A mutation in *argP* (transcriptional regulator of *argO*, which encodes for an arginine exporter) was identified as a potential candidate for improved arginine production. The combination of multicopy of *argP216* or *argO* and *argA214* led to nearly 2-fold and 3-fold increase in arginine production, respectively, and a reduction of acetate formation.

**Conclusions:**

In this study, *E. coli* was successfully engineered for enhanced arginine production. The *∆adiA*, *∆speC*, *∆speF*, *∆argR*, *∆argA* mutant with high gene copy number of *argA214* and *argO* produced 11.64 g/L of arginine in batch fermentation, thereby demonstrating the potential of *E. coli* as an industrial producer of arginine.

## Background

L-arginine has gained considerable interest from the pharmaceutical industry, notably because it is a precursor to nitric oxide, a blood vessel dilator [1]. Arginine is also commonly used in cosmetics, dental care products, dietary supplements and flavoring agents. It has also recently been shown that arginine can be used as an efficient nitrogen source and a potential alternative to inorganic nitrogen in plant fertilizers [2].

Given the wide utilization of arginine, there is a significant industrial demand for this amino acid, especially from sources that can guarantee an environmentally and economically sustainable production. Biotechnology processes, encompassing microbial biosynthesis, for the production of arginine from renewable resources need to be further explored to enable an industrial vital production. L-arginine can be synthesized *de novo* from L-glutamate by a large group of microorganisms. Members of the genus *Corynebacterium* are well-known L-glutamic acid producers [3] and are widely used for commercial production of amino acids [4], therefore they have been the organism of choice for microbial L-arginine production [5]. Recently, Park et al. [6] engineered a *C. glutamicum* strain able to produce 92.5 g/L L-arginine during fed-batch fermentation in 5 L bioreactors. However, other organisms can also be considered as arginine producers; for a review on the production of L-arginine by different microorganisms, including members of the *Corynebacterium* family, see [7]. Favorable traits of *Escherichia coli*, such as fast growth in inexpensive media, robust organism for industrial processes, and its well characterized metabolism and available molecular tools for genetic engineering, render it an organism of interest for the production of arginine. A number of patents regarding arginine production by *E. coli* strains exist (e.g. [8-10]). The inventors claim to have obtained up to 19.3 g/L in batch-fermentations with an acetate utilizing mutant derived from an arginine producing *E. coli* strain [8] and 11.6 g/L in 2 mL test tubes by attenuating the expression of genes encoding the lysine/arginine/ornithine ABC transporter [9]. However, the literature concerning *E. coli* and arginine biosynthesis has mainly been focused on the genetics and regulation systems rather than the production. To the best of our knowledge, no study has been published on the fermentative production of arginine by *E. coli*.

**Figure 1:**
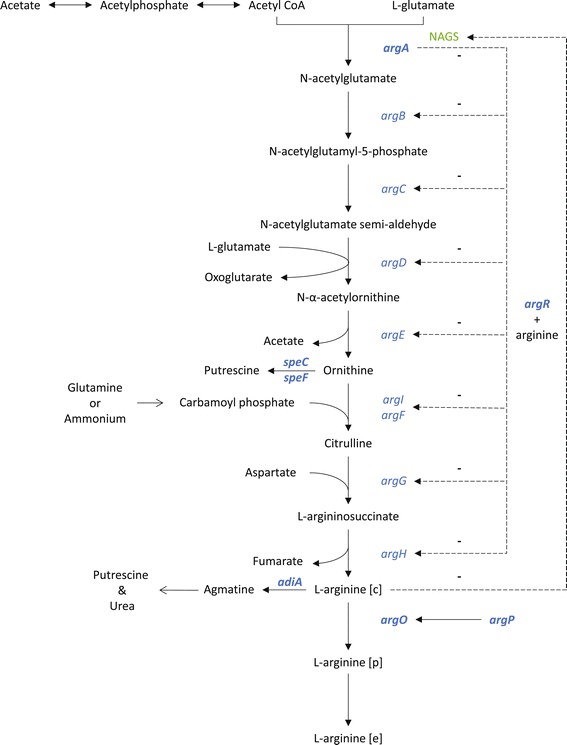
**Arginine biosynthesis pathway in** ***Escherichia coli*** **.** NAGS: N-acetylglutamate synthase, −: inhibition/negative regulation, [c]: cytoplasm, [p]: periplasm, [e]: extracellular. Targeted genes are indicated in bold.

In *E. coli*, arginine biosynthesis follows a linear pathway starting from the precursors glutamate and acetyl-CoA (Figure 1). The first enzyme in the biosynthetic pathway, *N*-acetylglutamate synthase (NAGS) encoded by the *argA* gene, is inhibited by arginine through feedback inhibition [11]. In addition, the arginine responsive repressor protein ArgR, encoded by the *argR* gene, negatively regulates transcription of arginine biosynthesis genes [12]. *E. coli* also possesses machineries for the export of some amino acids, including arginine. The arginine export pump ArgO, encoded by the *argO* gene, is transcriptionally regulated by ArgP [13-15]. The latter is responsive to intracellular arginine levels and activates the transcription of *argO* accordingly [15-17]. In addition, *E. coli* has degradative pathways for both L-arginine and its precursor L-ornithine. Two ornithine decarboxylases (encoded by the *speC* and *speF* genes) are responsible for the conversion of ornithine to putrescine [18,19], whereas arginine is first degraded into agmatine by an arginine decarboxylase (encoded by *adiA*), which is subsequently converted to putrescine and urea [18]. In summary, the biosynthesis pathway of arginine is constrained by several layers of metabolic and transcriptional regulations resulting in a complex network to engineer for arginine overproduction. Arginine overproducing *E. coli* strains have been classically obtained by selection of canavanine-resistant mutants [20]. Canavanine, an arginine analogue, inhibits growth by competing for arginine in protein synthesis [21]. The rationale for using this selection system is that mutants resistant to canavanine are likely to be derepressed for arginine synthesis, as over-production of arginine will release the inhibition caused by canavanine. When characterized, these mutants have subsequently been found to carry mutations in *argA*, *argR* and in some instances *argP* [12,22,23]. Not surprisingly, mutations in *argA* commonly resulted in an ArgA feedback resistant to arginine, which led some workers to derive further mutants by directed selection [24]. Similarly, mutations in *argP* resulted in ArgP acting in a constitutive manner, independent of the presence of arginine [14,17].

In this study we used both rational design based on known regulatory and metabolic information and selection procedures aiming for *E. coli* strains with enhanced production of arginine. Experimental validation of the engineered strains was carried out in 1-L fermenters under controlled conditions. We examined first the impact of deleting the *speC*, *speF*, *adiA* and *argR* genes and introducing a feedback-resistant *argA*. Then, the wild type *argA* was knocked-out and the feedback-resistant variant overexpressed. Finally, arginine production was significantly increased by overexpression of either *argP* or *argO*.

## Results & discussion

### Effect of introduction of feedback resistant variants of *argA* and selection of glutamate producing strains

In the first rate-limiting step of the arginine synthesis, NAGS, encoded by *argA*, catalyzes the acetylation of glutamate. To block the feedback inhibition of NAGS the plasmids pKH15 and pKH19, derived from the ASKA- plasmid pCA24N, were transferred into C600^+^Δ4 (see Table 1) to over-express the feedback resistant variants of *argA* (H15Y for *argA214* and Y19C for *argA215*) under the control of an IPTG-inducible promoter [25] (Table 1). The strain C600^+^Δ4 carrying either plasmid pKH15 or pKH19 could not be grown on M9 minimal media containing IPTG without exogenous glutamate supplementation and only weak growth was observed on the same medium when both glutamate and IPTG were absent. This suggested that over-expression of the feedback resistant *argA* in this strain resulted in glutamate starvation. To overcome this limitation, spontaneous mutants able to grow in the absence of glutamate were selected by plating washed and diluted cell cultures on M9 medium supplemented with IPTG without glutamate. Twelve colonies were picked at random and screened for arginine production based on the bioassay method. The three clones with the highest arginine production were chosen for subsequent work (SJB001, 003 and 004).

**Table 1:** **Plasmids and strains used in this study**

**Plasmid/Strain**	**Relevant characteristics/genotype**	**Source/Reference**
**Plasmids**		
pKH15	pCA24N (clone JW2786), *argA214*	ASKA- collection [26], this work
pKH19	pCA24N (clone JW2786), *argA215*	ASKA- collection [26], this work
pTrc99a	Amp-R, *lacIq*	Lab stock
pTrcArgP216	pTrc99a with a mutant *argP216* allele	This work
pJB044	pBR322 derived, *infA, rop-*	Lab stock [27]
pJB044argA15	pJB044 with *argA214* downstream of *infA*	This work
pJB044p1argA15	Same as pJB044argA15 but with *rrsBp1*	This work
pArgObla	Arginine bio-sensor plasmid with *bla* (Amp-R) under transcriptional control of *argOp*	This work
pArgObla10C	Arginine bio-sensor plasmid with *bla* (Amp-R) under transcriptional control of *argOp* with mutation in RBS	This work
pTrcArgP216	pTrc99a with *argP216* cloned under transcriptional control of trc promoter	This work
pJB044argAO	pJB044argA15 with *argO* cloned downstream of *argA214*	This work
pJB044argAP	pJB044argA15 with *argp216* cloned downstream of *argA214*	This work
**Strains**		
*E. coli* K-12 C600	*thr-,1 leuB6, thi-1, lacY1, glnV44, supE44, rfbD1, mcrA1*	Lab stock [28]
MG1655	*ilvG*-, *rfb*-50, *rph*-1	Lab stock
C600^+^	Same as C600 but *thr* ^*+*^ *, leu* ^*+*^	This work
C600^+^∆4	Same as C600^+^ but *∆adiA, ∆speC, ∆speF, ∆argR*	This work
pTrcArgP216/C600^+^∆4	C600^+^ ∆4 with a mutant *argP216* allele	This work
JW3932	Auxotrophic for arginine *∆argH*	[29]
SJB001	Glutamate independent mutant of C600^+^∆4 with pKH15 (clone 2)	This work
SJB003	Glutamate independent mutant of C600^+^∆4 with pKH19 (clone 2)	This work
SJB004	Glutamate independent mutant of C600^+^∆4 with pKH19 (clone 4)	This work
SJB003A	SJB003 but no plasmid	This work
SJB005	SJB003A but *∆argA*	This work
SJB015	SJB005 with pJB044argA15	This work
SJB025	SJB005 with pJB044p1argA15	This work
SJB006	Arginine producing mutant of C600^+^∆4 from biosensor selection, *argP216*	This work
SJB007	Derivative of SJB006 from second round biosensor selection	This work
SJB009	SJB005 with pJB044argAO	This work
SJB010	SJB005 with pJB044argAP	This work

Although these three strains were constructed in the same way, fermentations revealed very different growth behavior and arginine production abilities (Table 2). Indeed, SJB003 produced more arginine, with a productivity of 0.14 g/L/h and a final arginine concentration (3.03 g/L) significantly higher than that of the other similar mutants. The higher arginine producing capability of SJB003 compared to that of SJB001and 004 indicates that this strain had acquired beneficial mutations during growth under glutamate limitation. SJB003 was therefore chosen as a chassis for further genetic manipulation, although its beneficial mutations were not characterized.

**Table 2:** **Comparison of the performances of the different** ***E. coli*** **strains for arginine production by fermentation**

***E. coli*** **strain**		**Yields**						
	**μ (1/h)**	**Y**_**X/S**_ **(g dcw/g glc)**	**Y**_**P/S**_ **(g arg/g glc)**	**Y**_**P/X**_ **(g arg/g dcw)**	**Q**_**P**_ **(g/L/h)**	**Arginine (g/L)**	**Acetic acid (g/L)**	**Ac/Arg (mol ac/mol arg)**
SJB001	0.14 ± 0.02	0.26 ± 0.02	0.03 ± 0.00	0.10 ± 0.00	0.08 ± 0.00	1.94 ± 0.12	5.57 ± 0.11	8.5 ± 0.36
SJB003	0.14 ± 0.00	0.27 ± 0.01	0.04 ± 0.01	0.15 ± 0.03	0.14 ± 0.02	3.03 ± 0.59	6.12 ± 1.14	6.43 ± 2.36
SJB004	0.13 ± 0.02	0.27 ± 0.01	0.03 ± 0.00	0.11 ± 0.00	0.09 ± 0.00	2.04 ± 0.00	6.15 ± 0.24	8.90 ± 0.36
SJB015	0.04 ± 0.00	0.11 ± 0.00	0.07 ± 0.00	0.50 ± 0.02	0.08 ± 0.00	4.11 ± 0.49	15.85 ± 1.60	11.37 ± 0.22
SJB006	0.17 ± 0.01	0.35 ± 0.01	0.03 ± 0.00	0.09 ± 0.00	0.11 ± 0.00	2.03 ± 0.05	6.24 ± 0.45	9.07 ± 0.42
SJB007	0.16 ± 0.02	0.36 ± 0.01	0.04 ± 0.00	0.11 ± 0.00	0.14 ± 0.00	2.74 ± 0.21	5.31 ± 1.73	5.90 ± 2.31
SJB009	0.04 ± 0.00	0.12 ± 0.01	0.17 ± 0.01	1.18 ± 0.01	0.24 ± 0.01	11.64 ± 0.75	14.56 ± 0.93	3.72 ± 0.48
SJB010	0.09 ± 0.00	0.25 ± 0.01	0.11 ± 0.00	0.44 ± 0.02	0.29 ± 0.01	7.95 ± 0.04	3.14 ± 0.87	1.17 ± 0.33

Aerobic batch fermentations were performed in 1 L bioreactors at 32°C and pH 7; the initial glucose concentration was 70 g/L. μ, growth rate; Y_X/S_, biomass yield vs. glucose; Y_P/S_, product yield vs. glucose; Y_P/X_, product yield vs. cell mass; Q_P_, volumetric productivity. Results are given as means ± standard deviations.

Control fermentations with the parent strain C600^+^ were also performed. This strain did not yield any arginine (data not shown), which confirmed that the arginine productions displayed by the other strains are the result of their genetic modifications.

### Effect of overexpression of a feedback resistant *argA* on arginine production

To avoid the use of IPTG in an industrial process, it is of interest to place the feedback resistant *argA* gene under a constitutive promoter. First the SJB003 was cured of the pKH19 plasmid, harboring *argA215*, by repeated streaking on Luria Agar (LA) medium without antibiotic, giving rise to SJB003A. The *argA214* allele was chosen as the *argA* variant to be introduced in the strain since we found this allele to be slightly better for arginine productivity in preliminary shake flask experiments (data not shown). To avoid potential recombination with the new *argA214* plasmid, the chromosomal copy of the wild type *argA* gene was deleted in the SJB003A strain, resulting in SJB005.

The feedback resistant *argA214* was cloned into a high copy number plasmid pJB044 downstream of the *infA* gene encoding the translation initiation factor IF1. pJB044 carries a tetracycline resistance gene that can be removed by homologous recombination due to the presence of direct repeats flanking the gene, as previously described [27]. The *argA214* gene was placed downstream of the ribosome binding site (RBS) (AGGAGG) either with or without a strong constitutive rRNA promoter (rrsBp1) upstream (Table 1). The *argA* start codon GUG was changed by site-directed mutagenesis to the more efficient AUG codon in both constructs, termed pJB044argA15 and pJB044p1argA15. Strain SJB005 was the host of the pJB044 derived plasmids, resulting in the two IPTG-independent strains SJB0015 and SJB025 that differ only by the absence or presence of a strong rRNA promoter upstream of the *argA214* gene respectively.

**Figure 2:**
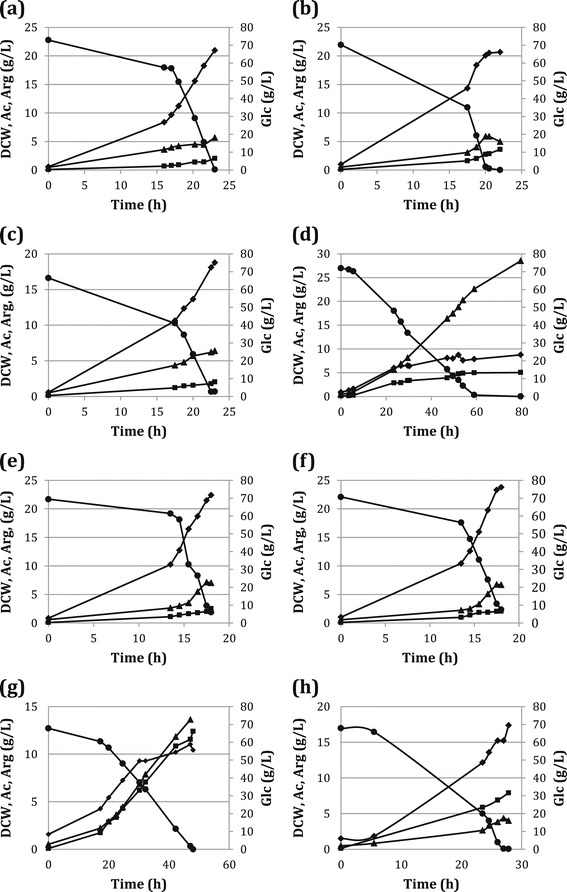
**Fermentation profile of (a) SJB001, (b) SJB003, (c) SJB004, (d) SJB015, (e) SJB006, (f) SJB007, (g) SJB009 and (h) SJB010.** ● glucose, ▲ acetic acid, ■ arginine and ◆ DCW.

When cultivated in bioreactors, SJB015 displayed a slightly improved arginine production compared to the previous constructs (Table 2). In particular, Y_P/x_ was relatively high (0.50 g arg/g dcw). However, cell growth was seriously hampered for this strain and SJB015 had the lowest μ and Y_X/S_ and the final cell density was lower than that of the other strains (data not shown). Consequently the volumetric productivity of SJB015 was relatively low (0.08 g/L/h). In addition, SJB015 produced high levels of acetate. When a DCW of about 7 g/L was reached (Figure 2d), cell growth stopped, arginine production drastically decreased and the remaining sugar (approximately 30% of the initial glucose) was mainly used for acetate formation (up to 28 g/L).

SJB025 exhibited slow growth on both rich (LA) and minimal medium (M9) with the appearance of some large colonies (data not shown). This suggested that the strong promoter driven *argA214* was toxic for the strain, with large colonies representing revertants. 50 of these colonies were screened for fast growing mutants with enhanced ability to produce arginine. However, none had retained this capacity (ascertained by the bioassay method) and consequently, neither these clones nor the parental SJB025 were used for further work.

For this strain it is likely that the rate of arginine formation exceeds the capacity of the arginine export system due to the overexpression of *argA214* in combination with the presence of a strong promoter upstream of *argA214*. The resulting accumulation of arginine inside the cell might have a variety of negative effects on cellular processes, which could explain why cell growth was seriously hampered in SJB025 and the ability for enhanced arginine production easily lost. This is consistent with previous reports, where mutants of *C. glutamicum* with deletion of *lysE*, encoding an exporter similar to ArgO [30,31]*,* were growth inhibited in the presence of intracellular arginine [30-32]. Growth arrest due to intracellular arginine in *∆argO* and *∆argP* mutants of *E. coli* has also been reported [14]. The increased nitrogen flow towards arginine production might also hinder the biosynthesis of other metabolites required for cell growth.

### Identification of novel mutations for enhanced arginine production using a biosensor

To complement the above described rational strain improvement strategies, a selection procedure was employed to select novel or previously unidentified mutants with increased arginine production. C600^+^Δ4 carrying the biosensor plasmid pArgObla10C was used for direct selection and screening of arginine accumulating mutants on M9 plates supplemented with 2, 3 and 4 mg/mL Amp. Only mutants with increased expression of the *bla* gene, most likely through increased transcription of the argOp promoter, can grow on media with Amp concentration higher than 0.6 mg/mL.

High Amp resistant mutants were randomly chosen from each Amp concentration and assayed for arginine production using the bioassay method. The isolated mutant showing the highest production was cured of the plasmid by repeated streaking on M9 plates without antibiotic (resulting in SJB006). After the biosensor plasmid removal an improved arginine production was retained, indicating that the acquired increased arginine accumulation was due to chromosomal mutations. Chromosomal sequencing of *argA* and *argP* genes in this clone revealed wild type *argA* and a T647C mutation in *argP* resulting in a valine to alanine mutation in position 216 (V216A).

To assess the effect of the V216A mutation on arginine production the mutant allele *argP216* was cloned into a high copy number plasmid downstream of an IPTG inducible promoter (pTrc99a) to give pTrcArgP216. Even without IPTG induction, the arginine accumulation of the strain pTrcArgP216 / C600^+^Δ4 was equivalent to SJB006, as based on the bioassay method (data not shown). We thus concluded that the increase in arginine accumulation observed in SJB006 was at least partly due to the presence of the *argP216* allele.

Selection of mutants with increased arginine accumulation was extended by transforming SJB006 with pArgObla10C anew, and screening on LA plates supplemented with 6, 8 and 10 mg/mL of Amp. Several colonies were assayed for arginine production; the best clone was cured of the biosensor plasmid and used for further work (SJB007). Sequencing of *argA* and *argP* showed that SJB007 also carried wild-type *argA* and no other mutation on the *argP* gene, other than the V216A mutation present in the parent strain SJB006.

Even with only the wild type *argA*, SJB006 produced similar amounts of arginine as SJB001 and SJB004 during fermentation (Table 2). Further, the productivity of SJB006 (0.11 g/L/h) was even slightly higher due to a faster growth. SJB007, which results from a second level biosensor selection, displayed increased arginine production compare to its parent SJB006. This demonstrates the potential effects of the mutation V216A carried by these two strains on the *argP* gene, but also that there might be some additional unknown mutation in SJB007 promoting arginine production.

### Effect of co-overexpression of a feedback resistant *argA* and *argP* or *argO* on arginine production

The mutant allele *argP216* resulted in increased accumulation of arginine. Amongst other physiological functions in the cells, ArgP also controls the transcription of *argO.* It was therefore of interest to combine overexpression of each of these two genes with the known feedback resistant *argA214* allele.

The plasmid pJB044argAP was constructed such that the *argP216* allele was placed downstream of *argA214*, under the control of the RBS sequence AGGAGG. The plasmid pJB044argAO was constructed by placing an *argO* ORF with the RBS sequence AGGAGG, downstream of *argA214*. In addition the inefficient start codon GUG was changed for the canonical AUG. The plasmids pJB044argAO and pJB044argAP were transferred to SJB005, to yield SBJ009 and SJB010 respectively.

Slow growth was also observed in the strains having a gene involved in arginine transport overexpressed in combination with the *argA214* allele (Figure 2d, g and h). In particular, SJB009 had almost the same μ and Y_X/S_ as SJB015 and also produced significant amounts of acetate. Nevertheless, cells grew to a somewhat higher density and arginine was steadily formed throughout the whole fermentation. Furthermore, SJB009 had the highest arginine production per amount of cells (1.18 g arg/g DCW), 2 to 13-fold that of the other strains. Consequently this strain yielded the highest final arginine titer (11.64 g/L) at a fair production rate (0.24 g/L/h). Also, a low growth associated with a high Y_P/X_ means that a large part of the glucose is used for arginine formation. SJB009 therefore showed the highest Y_P/S_ (0.17 g arg/g glc) of all strains evaluated. ArgO is directly responsible for the transport of arginine outside the cytoplasm and the high Y_P/S_ might be the result of an immediate excretion, enhanced by ArgO, of the large amount of arginine produced, due to *argA214*.

Interestingly, despite overexpression of the *argP* gene, responsible for *argO* transcription, SJB010 had significantly lower product yields than SJB009, yet higher than the other mutants. However, the cells of this strain grew twice as fast as cells of SJB009 and therefore SJB010 had the highest productivity of all strains (0.29 g/L/h). Unlike SJB015 and SJB009, SJB010 did not form high levels of acetate but produced both acetate and succinate (4–5 g/L).

SJB009 and SJB010 are similar to SJB015 except that one of their genes responsible of arginine export has been altered (*argO* and *argP*, respectively). This resulted in an important increase in the final arginine concentration (+183% and +93%), productivity (+200% and +262%) and product yield Y_P/S_ (+143% and +57%) for SJB009 and SJB010, respectively, compared to SJB015. This positive effect of *argO* and *argP* overexpression has previously been observed, showing that *E. coli* strains carrying multicopy *yggA*^*+*^ (*argO*) and *argP*^*d*^ (S94L mutation) had a greatly increased arginine production as determined from cross-feeding ability on agar plate [14]. Export has been identified as the rate-limiting step for the production of different amino acids when using *C. glutamicum* [33-35]. Similarly, it seems that the arginine export system plays a major role for the arginine production by *E. coli*.

### Formation of acetate during arginine fermentation

All mutants produced acetate as the main by-product. Acetate is formed during the 5^th^ step of L-arginine biosynthesis from L-glutamate (Figure 1). However, for most strains the ratio of ac:arg produced was higher than 1:1 (Table 2), which means that acetate was also formed via another pathway.

The accumulation of acetate by *E. coli*, even in aerobic environment, when growing under conditions of high glucose consumption is known as overflow metabolism. It occurs when the rate of glucose consumption is greater than the capacity of the cell to reoxidize the reduced equivalents, i.e. NAD(P)H, generated by glycolysis. Instead of entering the tricarboxylic acid (TCA) cycle, the carbon flux from acetyl-CoA is diverted to acetate, likely to prevent any further NAD(P)H accumulation as only ATP is formed during acetate formation while the TCA cycle generates several reducing equivalents [36,37].

As fermentations were run in batch mode with high initial glucose concentration (70 g/L) overflow metabolism is to be expected. The acetate production depends on the specific glucose uptake rate, with acetate formation occurring only after a certain threshold [36]. SJB001;3;4;6 and 7 indeed produced large amounts of acetate compared to arginine, which allowed them to have a high glucose uptake (0.44 to 0.54 mol glc/mol dcw/h) and a fast growth (μ > 0.13 h^−1^). SJB009 and SJB010 however had a considerably lower ac/arg ratio, i.e. 3.72 and 1.17 mol/mol, respectively, compared to at least 5.9 mol/mol for the other strains. The glucose uptake was also reduced (0.33 and 0.36 mol glc/mol dcw/h) as well as the growth (μ < 0.09 h^−1^). It is possible that the redirection of carbon and nitrogen toward arginine results in a shortage of other essential amino acids, thereby limiting the growth and the need for fast glucose assimilation. This could also be because the carbon flow from acetyl-CoA is forced toward arginine biosynthesis by the overexpressed *argA214*, thereby limiting the formation of acetate from acetyl-CoA.

However SJB015 had the highest ac/arg ratio (11.37 mol/mol) despite having a low specific glucose uptake (0.36 mol glc/mol dcw/h). This strain produced 15.85 g/L of acetate, which is comparable to the 14.56 g/L produced by SJB009. It is therefore likely that a large part of the acetate produced by SJB015 comes from the increased carbon flux through the arginine pathway, but that mainly acetate was excreted while arginine accumulated inside the cell due to the export limitation.

## Conclusion

We reported the development *E. coli* strains overproducing arginine, by targeting genes regulating repression of arginine biosynthesis and competing degradation pathways in addition to amplification of genes for N-acetylglutamate formation and arginine export. The two final strains obtained (SJB009 and SJB010) had the highest arginine yield (1.18 and 0.44 g arg/g glc, respectively) and productivity (0.24 and 0.29 g arg/L/h, respectively) and will be used for further genetic improvement and/or process optimization. The fermentation process developed for the comparison of the different constructs needs to be further optimized regarding fermentation medium, process conditions and process control.

## Materials and methods

### Strain construction

#### Bacterial strains and plasmids

Table 1 lists the plasmids and bacterial strains used in this study. In addition, the *E. coli* strain DH5α was used as a primary host for all cloning work.

#### Cultivation media

Strains were grown for 24–48 h in 15 mL tubes at 37°C and 220 rpm on Luria Bertani (LB) or in M9 minimal media supplemented with thiamine 5 μg/mL and with the addition of 1.5% agar when solid media was used. When necessary, glutamate was supplemented at 0.8 mM. Unless otherwise indicated, antibiotics were used at the following concentrations: tetracycline 20 μg/mL; chloramphenicol 50 μg/mL; kanamycin 50 μg/mL; ampicillin (Amp) 200 μg/mL. For induction experiments, IPTG was used at a final concentration of 0.1 mM.

#### DNA manipulation

Cloning of genes and regulatory elements was achieved either through standard restriction enzyme based cloning methods, or with the use of the sequence- and ligation- independent cloning method [24]. Site directed mutagenesis was used in order to introduce single nucleotide alterations into plasmids [38]. All gene deletions were done according to the in-frame gene excision method described by Link et al. [39]. Deletions were verified by PCR and DNA sequencing.

#### Construction of a base strain for arginine production (C600^+^Δ4)

A base strain with deletions of key genes involved in the arginine and ornithine degradation pathways and the arginine repression system was first constructed. The threonine and leucine auxotrophic *E. coli* strain K-12 C600 [18] was used as a starting strain. C600 was made autotrophic by moving wild type alleles from MG1655 donor strain via P1 transduction and subsequent selection on M9 plates without any amino acids. The resultant strain was termed C600^+^. One target for increased arginine production was inactivation of the arginine repressor *argR* and genes in the arginine and ornithine degradation pathways. The genes *argR*, *adiA*, *speC* and *speF* were subsequently deleted in order to obtain the strain termed C600^+^Δ4.

#### Biosensor plasmid construction

To select for arginine producing mutants with previously unidentified mutations, a biosensor plasmid termed pArgObla, was engineered based on one previously described [40]. The arginine sensitive promoter argOp was used to control the transcription of the ampicillin resistance gene *bla*. In addition, the T1T2 terminator was placed upstream to prevent any transcription from upstream promoters. Plasmid sensitivity to arginine was decreased by mutating the core RBS AAGGA upstream of the *bla* gene to ACGGA to reduce the efficiency of the translation initiation. The Amp minimum inhibitory concentration was considerably reduced for C600^+^Δ4 carrying this new biosensor variant pArgObla10C, compared to pArgObla (from > 5 mg/mL to 0.6 mg/mL).

#### Bioassay for screening of arginine producing mutants

Spent M9 growth media from cultures of candidate arginine producers were separated from the cell biomass by centrifugation at 10500 × *g* for 5 minutes. The supernatant was then sterilized with a 0.2 *μ*m filter and diluted into the assay medium, i.e. M9 supplemented with Kan. The strain JW3932, which was obtained from the Keio collection [29], is auxotrophic for arginine (*ΔargH)*. This strain was inoculated into the assay medium and its growth (measured as OD_590_) was used to determine the arginine concentration, based on a reference curve constructed from known concentrations of arginine.

### Fermentations

#### Seed cultures preparation

Cultures of the strains constructed as described above were stored in LB at −80°C as 15% glycerol stocks. The medium used for cultivation of the strains consisted of (per liter): 70 g glucose, 15 g corn steep liquor, 15 g (NH_4_)_2_SO_4_, 1 g KH_2_PO_4_, 0.5 g MgSO_4_ · 7 H_2_O, 20 mg FeSO_4_ · 7 H_2_O, 12 mg MnSO_4_ · H_2_O, 0.5 mg thiamine · HCl and appropriate concentrations of antibiotics. 1 mL of the stock culture was inoculated to a 500-mL shake flask containing 100 mL of sterile medium and 4 g CaCO_3_ to maintain a pH of about 7. The seed culture was incubated at 32°C in an orbital shaker at 200 rpm until an optical density at 562 nm (OD_562_) between 1.6 and 2.5 was reached.

#### Fermentations

Batch fermentations were conducted in 1 L bioreactors (Biobundle 1 L, Applikon Biotechnology, the Netherlands). The medium used was the same as described above, supplemented with 0.4 g/L of antifoam. 100 mL of seed culture were added in a sterile bioreactor containing 600 mL of medium. Prior to inoculation, pH was adjusted to 7 and thereafter maintained at that pH by automatic addition of NH_3_ solution (14-15% v/v). Fermentations were performed at 32°C and initial stirring was set at 500 rpm. The dissolved oxygen concentration (DO) was controlled with pulses of air (at 10 vvm) and to maintain a DO level above 30% the stirring was gradually increased from 500 rpm to 1000 rpm. All fermentations were carried out in duplicates.

Samples were regularly taken throughout the fermentations for analysis of optical density, glucose, organic acids and arginine concentrations.

#### Cell growth measurement

Cell growth was estimated by measuring the OD_562_ of appropriately diluted samples. Dry cell weight (DCW, in g/L) was determined from the optical density using a linear relationship between OD_562_ and DCW established in our laboratory under similar conditions: DCW = 0.4507 × OD562 + 0.6095.

#### Glucose and organic acids analysis

The samples were centrifuged for 10 min at 10621 × *g* and 4*°*C. The supernatant was then diluted five times with water and filtered through a 0.2 *μ*m syringe filter. Quantification of glucose and organic acids was performed by HPLC using a guard column (Micro-Guard IG Cation H Cartridge, BioRad), a cation exchange column (Aminex HPX87-H, BioRad) and a Series 200 refractive index detector (PerkinElmer). The column was kept at 65*°*C and 0.005 M H_2_SO_4_ at a flow rate of 0.6 mL/min was used as mobile phase.

#### Arginine analysis

Samples were centrifuged at 10621 × *g* for 10 min at 4*°*C and appropriately diluted with water. Arginine concentration was measured using the UPLC-AccQTag method (UPLC Amino Acid Analysis System Solution, Waters). The amino acids were first derivatized using AccQ · Fluor™ UltraReagent, then eluted with AccQ · Tag Ultra Eluents A and B and separated on a bridged ethyl hybrid C_18_ column (AccQ · Tag Ultra Column, 2.1 × 100 mm, 1.7 μm, Waters) with UV detection at 260 nm (ACQUITY UPLC System, Waters).
